# Capturing subjective cognitive decline with a new combined index in low education patients with Parkinson’s disease

**DOI:** 10.3389/fneur.2024.1403105

**Published:** 2024-08-19

**Authors:** Juan Huang, Hui Wang, Lin Chen, Binbin Hu, Xin Qin, Qiushuang Yang, Yajing Cui, Shenjian Chen, Wei Huang

**Affiliations:** Department of Neurology, The Second Affiliated Hospital, Jiangxi Medical College, Nanchang University, Nanchang, China

**Keywords:** Parkinson’s disease, subjective cognitive decline, low education, dysfunction in executive abilities/attention/language, new combined index

## Abstract

**Objectives:**

Subjective Cognitive Decline (SCD) refers to self-reported cognitive decline with normal global cognition. This study aimed to capture SCD among low educated patients with Parkinson’s disease (PD) using a newly established indicator.

**Methods:**

We recruited 64 PD patients with low education levels (education ≤12 years) for the study. The presence of SCD was determined based on a Unified Parkinson’s Disease Rating Scale Part I (1.1) score ≥ 1. Spearman analysis and multivariate binary logistic regression analyses were conducted to investigate factors associated with the PD-SCD group. The receiver operating characteristic (ROC) curve was used to evaluate the sensitivity and specificity of the new combined index.

**Results:**

The prevalence of SCD in PD patients was 43.75%. Low educated PD-SCD patients had higher scores on the Non-Motor Symptoms Scale (NMSS), Parkinson’s Fatigue Scale (PFS), Epworth Sleepiness Scale (ESS), as well as higher scores on the UPDRS-I and UPDRS-II, compared to PD patients without SCD. They also demonstrated poorer performance on the Montreal Cognitive Assessment (MoCA), particularly in the domains of executive abilities/attention/language. Multivariate binary regression confirmed the significant association between PD-SCD and MoCA-executive abilities/attention/language. Based on these findings, a combined index was established by summing the scores of MoCA-executive abilities, MoCA-attention, and MoCA-language. ROC analysis showed that the combined index could differentiate PD-SCD patients with an area under the curve (AUC) of 0.876. A score of 12 or less on the combined index had a sensitivity of 73.9% and a specificity of 76.2% for diagnosing PD-SCD.

**Conclusion:**

These low education patients with PD-SCD may exhibit potential PD-related pathological changes. It is important for clinicians to identify PD-SCD patients as early as possible. The newly combined index can help capture these low educated PD-SCD patients, with an AUC of 0.867, and is expected to assist clinicians in earlier identification and better management of PD patients.

## 1 Introduction

According to data released by the World Health Organization (WHO), the aging population is exacerbating cognitive deterioration, with 55.2 million people currently diagnosed with dementia (1). The projection suggested that by 2030, the number of affected people may rise to 78 million, resulting in a significant decrease in quality of life and a substantial increase in the socioeconomic burden. Parkinson’s disease (PD) is the second most common neurodegenerative disease globally, characterized by symptoms such as rest tremor, rigidity, postural instability, and bradykinesia (2). However, researchers have started shifting their focus to investigate the non-motor symptoms associated with PD, shedding light on a broader range of issues related to the condition (3). Notably, a review has demonstrated strong evidence that those with PD have a 6-fold higher chance of developing dementia than elderly people in a healthy condition (4). Furthermore, another review has indicated that cognitive function in individuals with PD progresses through three stages, similar to those seen in people with Alzheimer’s disease (AD), starting from subjective cognitive decline (SCD) and mild cognitive impairment (MCI) and advancing to dementia (PDD) (5).

In 1982, Reisberg (6) first proposed a general concept of SCD to describe self-reported cognitive decline with normal global cognition (7). It is worthy of note that PD-SCD, with a prevalence ranging from 30.3% to 85% over the disease, has the potential to predict future PD-MCI with considerable accuracy (8). It is estimated that around 40% of individuals with PD will develop PD-MCI at some point during the course of their illness (9). A 4-year longitudinal study conducted by Janvin et al. demonstrated that approximately 62% of PD-MCI patients eventually progressed to dementia, and a meta-analysis also highlighted MCI as an independent risk predictor for PDD (10, 11). As cognitive dysfunction advances into PDD, it gradually impairs an individual’s motor engagement and diminishes their quality of life. Given the prevalent occurrence of PD-MCI and the highly disabling nature of PDD, PD-SCD emerges as a potential precursor of PD-MCI, indicating the eventual development of PDD. As a risk state and an early warning sign for PD-MCI, PD-SCD should be used as a starting point for educating patients and their caregivers. Therefore, early identification of SCD among PD patients is of paramount importance.

The Montreal Cognitive Assessment (MoCA) scale and the Mini-Mental State Examination (MMSE) scale were the most commonly used objective scales to detect global cognitive impairment in PD (12). A meta-analysis indicated that the recommended cut-off score of 26/30 led to an increase in false positive test results, particularly among individuals with lower education levels (13, 14). In different cultures, the suggested MoCA cut-off scores for distinguishing MCI from normal cognition range between 13 and 26 (15). Moreover, it is been shown that low levels of education significantly increase the risk of developing PDD (12).

In this study, we used the recommended cutoff score of 27/30 of MMSE, and the developers of MMSE added a one-point correction for those with an education level ≤ 12 (16–18). Additionally, several studies used MMSE to evaluate the global cognitive function of PD-SCD patients (19–21). Thus, in our study, we aimed to (1) describe the prevalence of PD-SCD in low education patients; (2) find a new combined index to capture PD-SCD through the main domains of MoCA rather than a total score to reduce the high false-positive rate.

## 2 Materials and methods

### 2.1 Study patients

A consecutive inclusion of 64 patients from a movement disorder clinic was undertaken. A physical examination and a comprehensive assessment were conducted to obtain a full and detailed account of the patient’s condition. Age, gender, education level, and duration of disease were recorded. All of the patients were diagnosed with idiopathic PD according to the International Parkinson’s and Movement Disorder Society (MDS) clinical diagnostic criteria by two movement disorder disease specialists. Additionally, cognitive functioning was assessed by these specialists, who evaluated participants’ performance relative to estimates of premorbid ability. According to the guidelines for the diagnosis of dementia in PD published by the MDS, MMSE ≥27 was defined as an objective cognitive test normal (18). The following are the inclusion criteria: (1) those with an MMSE score ≥ 27, (2) those with an education level ≤ 12, (3) those who had the ability to collaborate with the researchers on their own, and (4) those who were able to communicate, write, and read. Patients were excluded based on the following criteria: (1) those with a prior diagnosis of dementia or MCI, (2) a diagnosis of secondary or atypical Parkinsonism, (3) those with brain disorders (e.g., cerebral infarction, cerebral hemorrhage, or brain tumor) or with other organic chronic comorbidities, and (4) those with serious psychiatric diseases [e.g., Hamilton Anxiety Scale (HAMA) ≥ 21 (22), Hamilton Depression Scale (HAMD) ≥ 17 (23), or obsessive-compulsive disorder]. This study was approved by the Research Ethics Committee of the Second Affiliated Hospital of Nanchang University.

### 2.2 Neuropsychological testing

The participants underwent a comprehensive neurocognitive assessment. Motor symptoms severity was evaluated using the Unified Parkinson’s Disease Rating Scale (MDS-UPDRS) and Hoehn and Yahr (H-Y) stage (24). Cognitive function was assessed using the MMSE and MoCA. Non-motor symptoms were evaluated by the Non-Motor Symptoms Scale (NMSS) (25). Mood disorders were evaluated using the HAMD and HAMA. Sleep disturbances were assessed using the Epworth Sleepiness Scale (ESS) (26), Pittsburgh Sleep Quality Index (PSQI) (27), and Rapid Eye Movement (REM) Sleep Behavior Disorder Questionnaire (RBD) (28). Fatigue was evaluated using the Parkinson’s Fatigue Scale (PFS-16) (29). Participants’ quality of life was assessed using the Parkinson’s Disease Questionnaire (PDQ-39) (30). Patients’ autonomic dysfunction and impairment were screened via the Scales for Outcomes in Parkinson’s Disease–Autonomic (SCOPA-AUT) (31). All assessments were performed while participants were in the “ON” state of PD medication.

### 2.3 Diagnostic criteria for SCD

In this study, we define SCD as self-reported cognitive decline with normal global cognition. We asked participants the question drawn from the UPDRSI 1.1 (Do you have memory/thinking impairment or/with disorientation and executive dysfunction?), and based on the score, the participants were divided into two groups: a score of ≥1 indicated those with subjective cognitive decline (PD with SCD) and a score of <1 indicated those without (PD without SCD) (21, 32).

### 2.4 Statistical analysis

Data were analyzed using SPSS (version 26; IBM), and all statistical assumptions were checked, with the significance level set at *p* < 0.05. First, a Kolmogorov–Smirnov test or Q-Q plot was employed to assess the normality of the data. Then for continuous variables that followed a normal distribution, the mean and standard deviation were used to represent the data, and the independent samples *t*-test was applied to assess differences between the two groups. For categorical data, a chi-square test was utilized. For samples that did not conform to a normal distribution, the median and quartiles were used for representation, and nonparametric (Kruskal–Wallis H and Mann–Whitney U) tests were implemented to check for group differences across those measures. The correlation between SCD and objective and subjective indexes was conducted by Spearman’s correlation. Furthermore, stepwise regression was used to find the best cutoff to differentiate the PD-SCD from those without. The odds ratio (OR) value and 95% confidence interval (95% CI) were reported, and the receiver operating characteristic (ROC) curve was used to evaluate the sensitivity and specificity of the prediction model.

## 3 Results

### 3.1 Demographic and clinical characteristics

A total of 64 PD patients were enrolled in the study, and when utilizing the MMSE cutoffs, 28 people were classified as SCD (43.75%). Table 1 shows the demographic and clinical characteristics. PD-SCD patients had significantly higher NMSS (*p* = 0.008), ESS (*p* = 0.0014), and PFS-16 (*p* = 0.002) scores than those without SCD. In addition, participants with SCD displayed a higher score in MDS-UPDRS-I (*p* = 0.015), MDS-UPDRS-II (*p* = 0.034), and the total MDS-UPDRS (*p* = 0.016). Furthermore, the PD-SCD had a significantly lower score whether in the total MoCA score (*p* = 0.007) or the domains of executive abilities (*p* = 0.003), attention (*p* < 0.001), and language (*p* = 0.001).

**Table 1:** Demographic and clinical characteristics in PD patients with and without SCD.

	PD without SCD	PD with SCD	*p* value
	*n* = 36 (56.25%)	*n* = 28 (43.75%)	
Age^#^	64.59 ± 8.62	64.36 ± 8.40	0.912
Sex (male/female)	28/8	17/11	0.138
Education (years)^&^	9.11 (8.00,11.00)	8.07 (7.00,9.75)	0.064
Disease duration (years)^#^	6.07 ± 4.26	4.52 ± 2.47	0.143
H-Y stage^#^	1.30 ± 0.47	1.32 ± 0.48	0.909
UPDRS-I^#^	4.46 ± 3.38	6.96 ± 3.47	**0.015**
UPDRS-II^#^	6.89 ± 4.79	9.43 ± 4.47	**0.034**
UPDRS-III^#^	19.58 ± 10.71	24.89 ± 12.77	0.075
UPDRS-total^#^	31.19 ± 15.51	41.29 ± 17.09	**0.016**
MMSE^#^	28.39 ± 1.24	28.32 ± 0.98	0.815
MoCA^#^	25.71 ± 2.49	23.04 ± 3.56	**0.007**
Executive abilities^&^	4.52 (4.00,5.00)	3.61 (3.00,4.00)	**0.003**
Naming^&^	2.90 (3.00,3.00)	2.78 (3.00,3.00)	0.430
Attention^&^	5.95 (6.00,6.00)	5.30 (5.00,6.00)	**0.000**
Language^&^	2.81 (3.00,3.00)	1.91 (1.00,3.00)	**0.001**
Abstraction^&^	1.52 (1.00,2.00)	1.52 (1.00,2.00)	0.723
Memory^&^	2.00 (0.50,3.50)	2.04 (1.00,3.00)	0.895
Orientation^&^	6.00 (6.00,6.00)	5.87 (6.00,6.00)	0.172
NMSS^#^	20.42 ± 22.62	27.93 ± 16.09	**0.008**
PFS-16^#^	27.97 ± 16.54	41.74 ± 17.70	**0.002**
ESS^#^	5.74 ± 4.79	8.89 ± 4.97	**0.014**
PQSI^#^	7.06 ± 3.67	8.79 ± 4.30	0.088
RBD-HK^&^	17.96 (4.00,29.50)	18.52 (8.00,25.00)	0.495
SCOPA-AUT^&^	24 (22.00,27.25)	27 (24.00.29.00)	0.083
PDQ-39^#^	15.81 ± 12.49	21.41 ± 17.00	0.174
HAMA^#^	6.31 ± 5.47	7.75 ± 5.37	0.295
HAMD^#^	4.39 ± 4.17	5.71 ± 4.20	0.213

^#^Mean ± SD; ^&^Median (1/4 quartile, 3/4 quartile); Statistically significant differences (*p* < 0.05) are shown in bold. SCD, Subjective cognitive decline; H&Y stage, Hoehn and Yahr stage; UPDRS, Unified Parkinson’s Disease Rating Scale; MoCA, Montreal Cognitive Assessment; PFS-16, Fatigue severity scale; NMSS, Non-motor symptoms scale; ESS, Epworth sleepiness scale; PQSI, Pittsburgh sleep quality index; RBD-HK, Rapid eye movement sleep behavior disorder-HK; SCOPA-AUT, Scales for outcomes in Parkinson’s disease—Autonomic; PDQ-39, 39-item of the Parkinson’s disease questionnaire; HAMA, Hamilton anxiety scale; HAMD, Hamilton depression scale.

### 3.2 Correlations

To explore the factors associated with SCD in PD patients, we first divided variables into three categories: demographic characteristics, subjective factors (NMSS, PFS-16, ESS, PQSI, RBD-HK, SCOPA-AUT, PDQ-39, HAMA, and HAMD) and objective factors (total MoCA and its domains, UPDRS-III). Through Spearman analysis, we found no significant correlation between demographic characteristics and PD patients with SCD. However, we observed an obvious positive correlation between the scores of NMSS, PFS, ESS, UPDRS-I, UPDRS-II, and PD patients with SCD (*p* < 0.05). Meanwhile, the negative correlation between SCD and the score of total MoCA as well as its domains of executive abilities, attention, and language was also found (*p* < 0.05) (Table 2). To further investigate these correlations, we performed partial correlation analysis by adjusting those subjective factors whose *p* value <0.05 mentioned above, and we observed that the negative correlation between SCD and the objective factors remained (Supplementary Table S1).

**Table 2:** Correlation between SCD and demographic characteristics, subjective factors as well as objective factors.

Variables	r	*p* value
Demographic characteristics		
Age	−0.034	0.789
Sex	0.185	0.143
Education	−0.233	0.063
Disease duration	−0.137	0.375
H-Y stage	0.030	0.830
Subjective factors		
NMSS	0.309	**0.014**
PFS	0.356	**0.004**
ESS	0.298	**0.019**
PQSI	0.213	0.091
RBD-HK	0.096	0.501
PDQ-39	0.158	0.253
SCOPA-AUT	0.224	0.083
HAMA	0.148	0.244
HAMD	0.171	0.178
UPDRS-I	0.341	**0.006**
UPDRS-II	0.291	**0.020**
Objective factors		
MMSE	−0.026	0.836
MoCA	−0.382	**0.011**
Executive abilities	−0.446	**0.002**
Naming	−0.120	0.436
Attention	−0.555	**0.000**
Language	−0.501	**0.001**
Abstraction	−0.054	0.727
Memory	0.020	0.897
Orientation	−0.208	0.174
UPDRS-III	0.236	0.061

Statistically significant differences (*p* < 0.05) are shown in bold.

### 3.3 Factors associated with SCD in multivariate binary logistic regressions

In the multivariate binary logistic regression analysis, we used the presence of SCD as dependent variable and included factors with a significant *p* value from the Spearman analysis as independent variables. The results showed that PD-SCD patients had significantly poorer performance in MoCA-executive abilities (OR = 0.06, *p* = 0.019), MoCA-attention (OR = 0.02, *p* = 0.021), and MoCA-language (OR = 0.03, *p* = 0.029) (Table 3).

**Table 3:** Factors associated with SCD in multivariate binary logistic regressions.

Variables	OR	95%CI	*p* value
Subjective factors			
PFS	1.00	0.93–1.10	0.846
NMSS	1.02	0.88–1.18	0.759
ESS	1.44	0.98–2.13	0.066
UPDRS-I	0.89	0.35–2.24	0.805
UPDRS-II	0.96	0.66–1.40	0.847
Objective factors			
MoCA-executive abilities	0.06	0.05–0.62	**0.019**
MoCA-attention	0.02	0.01–0.42	**0.021**
MoCA-language	0.03	0.02–0.67	**0.029**
MoCA	2.73	0.99–7.56	0.053

Statistically significant differences (*p* < 0.05) were shown in bold.

### 3.4 A new combined index to identify PD patients with SCD

**Figure 1:**
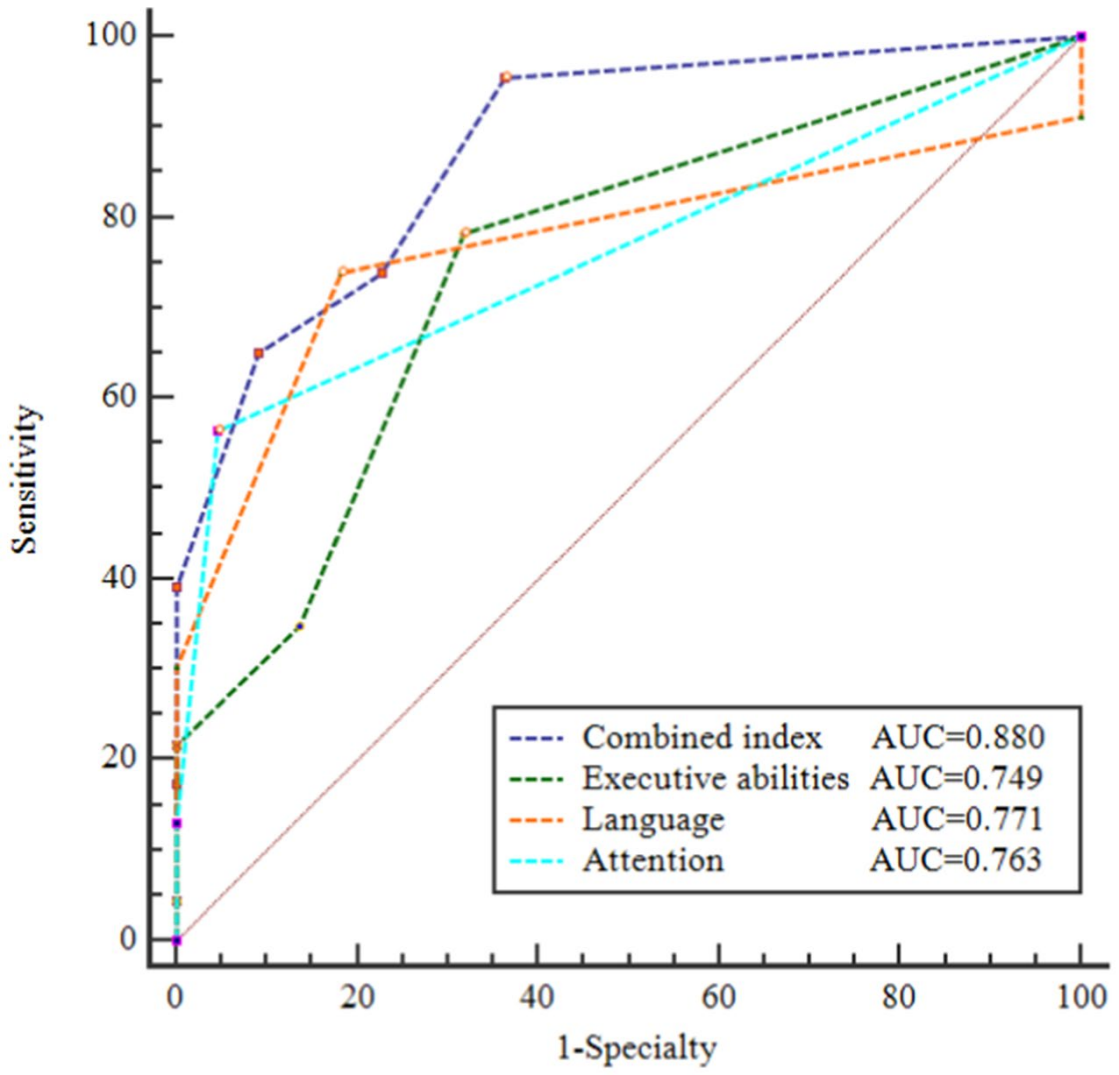
Receiver operating characteristic (ROC) curves of area under the curve (AUC) for discriminating PD patients with SCD from PD without SCD. AUC of these ROC curves were 0.880, 0.749, 0.771, and 0.763, respectively. Combined index = total scores of (MoCA-executive abilities + attention + language).

Based on the findings from logistic regression analysis, we discovered that MoCA subtests for executive abilities, attention, and language were significantly linked to PD-SCD, rather than the overall MoCA score. Thus, we try to find an objective index to help identify PD patients with SCD with low education levels. Subsequently, we established a new combined index by summing the scores of MoCA-executive abilities, MoCA-attention, and MoCA-language. As shown in Supplementary Table S2, the severity of the cognitive domain deficit was more significant in the patients with PD-SCD than in the patients without SCD: MoCA-executive abilities (*p* = 0.003), MoCA-attention (*p* < 0.001), MoCA-language (*p* = 0.001), and total scores of MoCA-executive abilities + attention + language (*p* < 0.001). The ROC was derived to quantify the area under the curve (AUC) which was an appropriate measure for describing model performance. The AUC of each classification is shown in Figure 1. An AUC of 0.876 demonstrated the efficacy of the composite index in distinguishing PD-SCD patients. The diagnosis of PD-SCD exhibited a 76.2% specificity and a 73.9% sensitivity for those with a combined index score of 12 or below.

## 4 Discussion

In our study, we focused on PD patients with low education levels and sought to understand the prevalence of SCD and its related factors in this PD population. As previous studies reported (13, 33, 34), we found a high false-positive rate for MCI when using the Milani et al. MoCA cutpoint. Rossetti et al. found that the mean MoCA score was 21 among those with less than 12 years of education, one-point educational adjustment is not enough for educational attainment (35). The cross-cultural applicability of the MoCA suggested cut-offs ranged from 13 to 26 to differentiate MCI from normal cognition (15). Therefore, we established a new combined index to distinguish low education level patients with SCD from those without SCD in the last, which may help us identify potential preclinical cognitive impairment in PD patients with low education as early as possible.

In this study, all participants had less than 12 years of schooling, reflecting the educational profile typical for their age group. This aligns with the criteria set by the developers of MMSE and MoCA, who consider 12 years as the threshold for lower education levels. We showed that 43.75% of PD patients subjectively reported SCD, which was higher than the value found in Xiao’s study and Siciliano’s study (32, 36). A study by Xiao showed that 22.3% of PD patients (*n* = 332) had SCD, but the sample had a younger age (56.3 years old) and a shorter disease duration (< 2 years) compared to our study. The younger age and shorter disease duration in Xiao’s study may contribute to a lower proportion of SCD, as studies have suggested that the prevalence of SCD increases with age and the duration of PD (37, 38). Siciliano’s study focused only on memory complaints and found that 15% of PD patients met the criteria for SCD on the Multifactorial Memory Questionnaire (MMQ). However, we should note that the “non-amnestic” pattern, attention, and executive dysfunction were more common in PD patients (39), which may contribute to a lower presence of SCD if only memory complaints are considered. Therefore, it is crucial to consider a broader range of cognitive impairments when assessing SCD in PD patients. We defined SCD as memory/thinking impairment or/with disorientation and executive dysfunction according to UPDRS-I 1.1. In our view, the subjective assessment tools used may also be responsible for the discrepancy here.

We also analyzed the association between SCD and various factors. Our results showed that the presence of SCD was associated with higher scores on NMSS, PFS, ESS, UPDRS-I, and UPDRS-II but did not differ in UPDRS-III, which was consistent with previous studies (32, 36, 40–42). NMSS were classified into nine relevant domains: cardiovascular; sleep/fatigue; mood/cognition; perceptual problems/hallucinations; attention/memory; gastrointestinal tract; sexual function; and miscellaneous (43). Previous studies reported a significant increase in NMSS scores among SCD patients, which may serve as a reminder that NMSS scores were associated with a risk of PD-SCD (9, 32, 40). A significantly higher PFS score in PD-SCD was supported by other studies (32, 36, 44). In Siciliano’s study, PD patients with fatigue had 5.97 times higher SCD than patients without fatigue, which revealed a possible shared pathologic mechanism between PD-SCD and fatigue.

Daytime sleepiness, as measured by the ESS, was found to be associated with cortical and subcortical brain atrophy, which can contribute to cognitive impairment (45, 46). Thus, a higher ESS score in the group of PD-SCD patients may indicate a potential PD-related pathological change. However, there were certain considerations to be considered when utilizing the ESS in our cohort of low-educated PD patients, such as the fact that this group of patients might not frequently read books, travel, or participate in social activities, therefore, some ESS questions may result in an inaccurate answer. Clinicians should closely monitor levels of sleepiness to ensure a more precise assessment of the relationship.

The UPDRS-I and UPDRS-II primarily focused on the daily living experiences of PD patients. Our results indicated higher scores on these scales in PD patients with SCD than without SCD, which was consistent with the findings of Rosenblum’s study (41). In Rosenblum’s study, significant differences were found in the Daily Living Questionnaire (DLQ) between PD with and without SCD. The study suggested the association between the DLQ and PD-SCD may be relevant for detecting subtle deficits in PD patients. Hence, these findings highlight the importance of monitoring subtle changes in daily activities when encountering PD patients with SCD.

Several studies (21, 47, 48) found an obvious connection between PD-SCD and anxiety, depression or apathy, but few studies debated the relationship between the main cognitive deficit and PD-SCD in low education patients. In our study, executive abilities, attention, and language were found to be the major contributing domains even after removing the influence of mood. These basic findings were consistent with research showing that PD-SCD performed poorer in attention-associated tasks and had difficulties with attention and executive function but not memory (49). In Mills et al.’s research, they suggested lower scores on executive functions in PD-SCD (50). In a longitudinal study conducted by Galiter et al., PD-SCD showed poor performance in the verb naming test, which suggested the possible linguistic dysfunction in PD-SCD (19). In a word, these above studies suggested a possible aberrant PD-related pathology in the PD-SCD group. Since the frontal lobe plays significant roles in executive functions, frontal-related cognitive dysfunction was thought to be associated with impairment of dopaminergic transmission to the frontal cortex. Attention seems to be associated with the parietal lobe as well as the anterior cingulate gyrus; attention in PD patients was associated with dopaminergic hypofunction in the caudate nucleus. The anterior cingulate gyrus is responsible for verbal fluency (51–55). However, there were discrepancies between our results and those of other studies. In Yang’s study, they reported memory dysfunction in PD patients with SCD compared to those without SCD (42). The difference may be attributed to a “non-amnestic” pattern in the PD group, and the tool used to measure memory in our study was too simple to find subtle memory dysfunction.

Similar to our research, a study concerning PD patients with subthreshold depression revealed decreased performance in subsets of the MoCA as well as an increase in subjective cognitive complaints (56). In a 3-year study, Mills divided MoCA into four domains and found a tendency for baseline MoCA memory to predict the degree of subjective decline (57). PD without SCD scored lower on the MoCA total score and the attention and working memory sub-score, according to a study that used the Parkinson’s Disease-Cognitive Functional Rating Scale (PD-CFRS) to evaluate the subjective cognitive symptoms (44). On the one hand, Mulligan’s study suggested that subjective report and objective cognitive performance in SCD individuals complement each other, and our findings have attempted to extend this work to the challenge of finding an objective index to assess SCD, especially in PD patients with low levels of education (58). On the other hand, compared to the total MoCA score, we found a stronger correlation between PD-SCD and executive ability/attention/language on the MoCA in patients with low education PD. Consequently, we developed a new combination index that proved to be a highly reliable objective measure (AUC = 0.867, sensitivity = 73.9%, specialty = 76.2%).

## 5 Limitations

This study has important strengths. We discussed the prevalence of SCD in individuals with poor levels of education, who have a risk for PDD. Although the developer increased one point, there were several shortcomings with the existing MoCA cutpoints for use with patients with low levels of education. To decrease the false-positive rate for MCI, we combined a new index. There are also several important limitations to our study. First, the sample in our study is expected to expand and longitudinal studies are essential to validating our cross-sectional results. However, this study is part of a longitudinal study, we are recruiting the follow-up of patients, and larger samples and the follow-up data would be useful to confirm these findings. Second, we used a short MMSE scale to measure normal cognitive status rather than a comprehensive neuropsychological test; studies using a formal neuropsychological test battery are needed to confirm our results. Then, the UPDRSI-1.1 we used to define SCD was too simple compared with a comprehensive scale. However, SCD measured by UPDRS-I 1.1 has been proven to be associated with the deterioration of cognitive functions in PD patients, which confirms the clinical significance of the simple tool (21, 32, 37, 41). Finally, besides the recruitment in one center, we do not have brain imaging in this study, and the study of the relationship between cognitive and neuroimaging is being investigated in another multicenter study.

## 6 Conclusion

In conclusion, our study showed that there was a high prevalence of SCD in low educated PD patients. In addition, within this subgroup, they had poorer performance in executive abilities, attention, and language domains. Therefore, early identification of patients with PD-SCD is critical for clinicians. Comprehensive cognitive evaluations are costly, timely and energy consuming for low education patients. As a result, a new combined index has been developed that is an objective indicator rather than relying on subjective tools. It is the sum of the MoCA executive function/attention/language scores and captures PD-SCD patients with low education with an AUC of 0.867. This index is expected to assist clinicians in better managing and early identification of patients with PD-SCD who are low educated.

## Data Availability

The raw data supporting the conclusions of this article will be made available by the authors.
