# Supplementary material for: Capturing subjective cognitive decline with a new combined index in low education patients with Parkinson’s disease
Source: Front Neurol. 2024 Aug 19;15:1403105. doi: 10.3389/fneur.2024.1403105 (PMC11367866; doi:10.3389/fneur.2024.1403105)
Supplement: Supplementary file 1 [file Table_1.docx]

**Table S1** Correlation between SCD and objective factors by the partial correlation analysis

| **Variables** | ***r*′** | ***p*-value** |
| --- | --- | --- |
| **Objective factors** |  |  |
| MMSE | 0.179 | 0.275 |
| MoCA | -0.420 | **0.008** |
| Executive abilities | -0.508 | **0.001** |
| Naming | -0.132 | 0.422 |
| Attention | -0.516 | **0.001** |
| Language | -0.381 | **0.017** |
| Abstraction | 0.053 | 0.747 |
| Memory | -0.061 | 0.712 |
| Orientation | -0.252 | 0.121 |
| UPDRS-III | 0.007 | 0.966 |

***Note: r*′:** The partial correlation analysis was conducted by controlling subjective factors (UPDRS-I, UPDRS-II, NMSS, PFS, ESS); Statistically significant differences (p < 0.05) are shown in bold.
