# Supplementary material for: Capturing subjective cognitive decline with a new combined index in low education patients with Parkinson’s disease
Source: Front Neurol. 2024 Aug 19;15:1403105. doi: 10.3389/fneur.2024.1403105 (PMC11367866; doi:10.3389/fneur.2024.1403105)
Supplement: Supplementary file 2 [file Table_2.docx]

**Table S2** The characteristics of new combined index in PD patients with and without SCD

| **Index** | **PD without SCD** | **PD with SCD** | ***p*-value** |
| --- | --- | --- | --- |
| MoCA-executive abilities | 4.52(4.00,5.00) | 3.61(3.00,4.00) | 0.003 |
| MoCA-attention | 5.95(6.00,6.00) | 5.30(5.00,6.00) | < 0.001 |
| MoCA-language | 2.81(3.00,3.00) | 1.91(1.00,3.00) | 0.001 |
| Combined index | 13.29 ± 1.06 | 10.83 ± 1.92 | <0.001 |

***Note:*** Combined index = total scores of (MoCA-executive abilities + attention + language)
